# L-Carnitine Prevents Progression of Non-Alcoholic Steatohepatitis in a Mouse Model with Upregulation of Mitochondrial Pathway

**DOI:** 10.1371/journal.pone.0100627

**Published:** 2014-07-01

**Authors:** Hisashi Ishikawa, Akinobu Takaki, Ryuichiro Tsuzaki, Tetsuya Yasunaka, Kazuko Koike, Yasuyuki Shimomura, Hiroyuki Seki, Hiroshi Matsushita, Yasuhiro Miyake, Fusao Ikeda, Hidenori Shiraha, Kazuhiro Nouso, Kazuhide Yamamoto

**Affiliations:** Department of Gastroenterology and Hepatology, Okayama University Graduate School of Medicine, Dentistry and Pharmaceutical Sciences, Okayama, Japan; University of Salento, Italy

## Abstract

Non-alcoholic steatohepatitis (NASH) is a severe form of non-alcoholic fatty liver disease characterized by lobular inflammation, hepatocellular ballooning, and fibrosis with an inherent risk for progression to cirrhosis and hepatocellular carcinoma (HCC). Mitochondrial dysfunction appears to play a role in the progression from simple steatosis to NASH. L-carnitine (L-b-hydroxy-g-N-trimethylaminobutyric acid), an essential nutrient that converts fat into energy in mitochondria, has been shown to ameliorate liver damage. The aim of the present study was to explore the preventive and therapeutic effect of L-carnitine in NASH model mice. Eight-week-old male STAM mice, a NASH-cirrhosis-hepatocarcinogenic model, were divided into 3 experimental groups and fed as follows: 1) high-fat diet (HFD) (control group); 2) HFD mixed with 0.28% L-carnitine (L-carnitine group); and 3) HFD mixed with 0.01% α-tocopherol (α-tocopherol group). After 4 or 8 weeks, mice were sacrificed. Blood samples and livers were collected, and hepatic tumors were counted and measured. Livers were subjected to histological study, immunohistochemical staining of 4-hydroxynonenal and ferritin, determination of 8-OHdG levels, mRNA and protein expressions for multiple genes, and metabolomic analysis. The intestinal microbiome was also analyzed. L-carnitine increased hepatic expression of genes related to long-chain fatty acid transport, mitochondrial β-oxidation, and antioxidant enzymes following suppression of hepatic oxidative stress markers and inflammatory cytokines in NASH, and mice treated with L-carnitine developed fewer liver tumors. Although α-tocopherol resulted in NASH improvement in the same manner as L-carnitine, it increased periodontitis-related microbiotic changes and hepatic iron transport-related gene expression and led to less effective for anti**-**hepatocarcinogenesis.

**Conclusion:**

L-carnitine prevents progression of non-alcoholic steatohepatitis in a mouse model by upregulating the mitochondrial β-oxidation and redox system.

## Introduction

Non-alcoholic fatty liver disease (NAFLD) is a common liver disease that is characterized by hepatic steatosis. Most patients with NAFLD exhibit non-progressive simple fatty liver, namely non-alcoholic fatty liver. Non-alcoholic steatohepatitis (NASH) is a more severe form of NAFLD that is characterized by lobular inflammation, hepatocellular ballooning, and fibrosis with an inherent risk for progression to cirrhosis and hepatocellular carcinoma (HCC) [1]. These features were defined by Ludwig et al. in 1980 to describe a liver disease that histologically mimics alcoholic hepatitis but occurs in individuals who do not abuse alcohol [2]. Because NASH can progress towards end-stage liver disease requiring a liver transplant, therapies for NASH must be developed. Even though the mechanisms of the progression from simple steatosis to NASH are not completely understood, accumulating evidence suggests a major role of mitochondrial dysfunction in steatosis and steatohepatitis [3]. Mitochondrial dysfunction not only impairs fat homeostasis in the liver but also leads to an overproduction of oxidative stress-inducing reactive oxygen species (ROS) that trigger lipid peroxidation, cytokine overproduction, and cell death. Indeed, ultrastructural alterations, impairment of adenosine triphosphate synthesis, and increased production of ROS have been reported in liver mitochondria from NASH patients and a rodent model [4], [5].

Vitamin E supplementation, the prototypical antioxidant drug treatment, has become a standard treatment for NASH [6]. However, most clinical studies of atherosclerotic diseases with dietary antioxidants have not generated clear results, partly because of the non-selective effects of these anti-oxidative drugs [7]. Controversy surrounds antioxidant therapies because ROS have essential functions in living organisms.

L-carnitine (L-b-hydroxy-g-N-trimethylaminobutyric acid) is an essential nutrient that converts fat into energy in mitochondria. It acts as a carrier for fatty acid across the mitochondrial membrane and is also present in the free or acyl-carnitine form in plasma [8], [9]. L-carnitine plays an important role in lipid metabolism; it acts as an essential cofactor for the β-oxidation of fatty acids by facilitating the transport of long-chain fatty acids (LCFAs). It can activate carnitine palmitoyltransferase (Cpt), the key enzyme in fatty acid oxidation [10]. Recently, L-carnitine has been proposed for the treatment of various diseases, including liver injury. Several studies have shown that L-carnitine administration can ameliorate or prevent liver damage of various etiologies. Animal studies have shown that dietary supplementation with L-carnitine prevents hepatitis and subsequent HCC [11]. L-carnitine is not an oxidative stress scavenger but it may stimulate mitochondrial function, and the effect would be different from typical antioxidants. The aim of the present study was to explore the preventive and therapeutic effects of L-carnitine in NASH model mice in order to provide evidence for L-carnitine as a treatment candidate for NASH.

## Materials and Methods

### Animals and experimental design

STAM mice, a NASH-cirrhosis-hepatocarcinogenic model, were purchased from Stelic Institute & Co., Inc. (Tokyo, Japan). This mouse model progresses from NAFLD to NASH at 8 weeks of age and develops HCC at 16 weeks of age [12]. Briefly, C57BL/6J male mice were injected with streptozotocin 2 days after birth to destroy pancreatic beta cells, and were fed a high-fat diet (HFD-32; CLEA-Japan, Tokyo, Japan).

For NASH experiments, 8-week-old male STAM mice were divided into three experimental groups and fed for 4 weeks as follows: 1) high-fat diet (HFD) (Control group); 2) HFD mixed with 0.28% L-carnitine (LKT Laboratories, Inc., St. Paul, MN, USA) (L-carnitine group); and 3) HFD mixed with 0.01% α-tocopherol (LKT Laboratories, Inc.) (α-tocopherol group). Diets were replaced 3 times per week to keep them dry, as high fat diets tend to retain moisture. After 4 weeks, mice were sacrificed. Blood samples were obtained from the right atrium by cardiac puncture, and the livers were excised. Livers were cut into pieces and fixed in 10% formalin for histological analysis, or fresh-frozen in liquid nitrogen and stored at −80°C in a freezer until use. L-carnitine and α-tocopherol concentrations were decided based on previous reports, and were confirmed to show the same improvement in histological activity and stage in our STAM mouse model [13], [14].

For hepatocarcinogenesis experiments, 8-week-old STAM mice were divided into three experimental groups and fed for 8 weeks as described above. After 8 weeks, mice were sacrificed and hepatic tumors were counted and measured.

Animals had free access to water and food and were maintained under specific pathogen-free (SPF) conditions in a temperature-controlled animal facility with a 12-h light-dark cycle. Cages housed 4 mice and the bedding material was made from paper rolls (Paper Clean; Peparlet Co., Ltd, Fujieda, Japan). All protocols and procedures conformed to the guidelines of the Okayama University Committee for Care and Use of Laboratory Animals and were approved by the Animal Experiments Ethics Committee of Okayama University.

### Measurement of weight, fasting blood glucose, plasma aminotransferases and triglycerides

Body weight of mice at sacrifice on week 12 after birth was a median 19.9 g (interquartile range; 18.0–21.2 g). Fasting blood glucose levels were measured with a blood glucose meter (GT-1820; Arkray Inc., Kyoto, Japan). Alanine aminotransferase and triglyceride levels were determined by standard methods at Skylight Biotech Company (Akita, Japan).

### Histological analysis of liver

Sections of formalin-fixed livers were stained with hematoxylin-eosin. NAFLD activity was assessed by NAFLD activity score, as described by Kleiner et al., with separate scores for steatosis (0–3), hepatocellular ballooning (0–2), and lobular inflammation (0–3). NAFLD activity score is the sum of these scores, and values of ≥5 are correlated with a diagnosis of NASH. All liver specimens were assessed by two hepatologists (TY and AT) blinded to the identities of the study groups.

### Immunohistochemistry of oxidative damage marker and ferritin

Immunohistochemical staining of 4-hydroxynonenal (4-HNE) and ferritin was performed. Liver samples were fixed in 10% formaldehyde. The sections were incubated with a 1∶100 dilution of primary antibody for 4-HNE (clone HNEJ-2; JaICA, Shizuoka, Japan) at 4°C overnight. Subsequently, they were incubated with secondary antibody (Dako: LSAB + System-HRP), universal biotinylated link antibody, and streptavidin-HRP. Slides were stained with fresh 3,3′-diaminobenzidine chromogen (Dako, Liquid diaminobenzidine + Substrate Chromogen System). For ferritin staining, primary antibody (Abcam, Cambridge, UK) was used at a 1∶1000 dilution. Quantitative comparison of immunohistochemistry staining was performed by computerized image analysis with Olympus cellSens imaging software (Olympus, Tokyo, Japan).

### Quantitative real-time polymerase chain reaction

Total RNA was prepared from liver tissue with TRIzol Reagent (Invitrogen, Carlsbad, CA, USA). Extracted RNA was converted into cDNA by reverse transcription (SuperScript III Reverse Transcriptase; Invitrogen). Specific gene expression was quantified by real-time PCR performed on a LightCycler 480 Instrument (Roche Diagnostics Ltd., Rotkreuz, Switzerland). Relative expression levels of target genes were compared after normalization against β-actin. Tumor necrosis factor (TNF)-α expression was determined with a LightCycler primer and probe set (Nihon Gene Research Laboratories, Sendai, Japan). The following genes were assessed with a SYBR green fluorophore: interleukin (IL)-1β, organic cation/carnitine transporter (OCTN) 2, Cpt1a, Cpt2, medium-chain acyl-CoA dehydrogenase (MCAD), superoxide dismutase (Sod)2, catalase (CAT), glutathione peroxidase (Gpx)1, Gpx4, divalent metal transporter 1 (DMT-1), and Hamp (hepcidin coding gene).

### Western blotting analysis

Total proteins were extracted from liver tissue using a protein extraction kit (Minute Protein Extraction Kit; Invent Biotechnologies, Inc., Eden Prairie, MN, USA). Extracted proteins were quantitated by the BCA method (BCA protein Assay Kit; Takara Bio, Inc., Otsu, Japan), and were diluted to the same protein concentrations. Equivalent amounts (50 µg) of each group were run on SDS polyacrylamide gels. Proteins were transferred electrically to a PVDF membrane and incubated with the following antibodies; anti-TNF-α, anti-IL1β (Cell Signaling Technology, Inc., Beverly, MA, USA), anti-DMT1, anti-hepcidine25, anti-CPT1A, anti-CPT2, anti-SOD2, anti-catalase (Abcam, Tokyo, Japan), anti-GPx4 (LifeSpan BioSciences, Seattle, WA, USA). Membranes were washed and incubated with respective secondary antibodies. Detection of bands was performed by chemiluminescent analysis. Beta actin was used as a loading control. Representative data of three experiments were shown on figures.

### Hepatic 8-hydroxydeoxyguanosine (8-OHdG) concentration analysis

8-OHdG, a modified DNA base product generated by free radicals, is a biomarker of oxidative DNA damage [15]. DNA was extracted from the liver using a DNA extractor kit (DNA Extractor TIS Kit; Wako, Osaka, Japan). Hepatic 8-OHdG concentration was measured by using an enzyme-linked immunosorbent assay kit (Highly sensitive 8-OHdG Check; Japan Institute for the Control of Aging, Shizuoka, Japan) after preparation with an exclusive kit (8-OHdG Assay Preparation Reagent Set; Wako). Results were expressed as ng/mg DNA corrected against the DNA level in each sample.

### Metabolomic analysis

For metabolomic analysis, control C57BL/6 mice fed a standard diet and STAM mice were compared. Eight-week-old male C57BL/6 mice were fed standard chow for 4 weeks as the BL6-Control group. The three experimental groups were STAM mice fed a HFD (control group); STAM mice fed a HFD mixed with 0.28% L-carnitine (L-carnitine group); and STAM mice fed a HFD mixed with 0.01% α-tocopherol (α-tocopherol group). After 4 weeks, liver tissue was harvested and immediately frozen in a −80°C freezer. The capillary electrophoresis time-of-flight mass spectrometry (CE-TOFMS)–based metabolome analysis was performed at Human Metabolome Technologies (Yamagata, Japan). Metabolites in the samples were identified by comparing the migration time and m/z ratio with authentic standards and quantified by comparing their peak areas with those of authentic standards.

### Intestinal microbiome analysis

Analysis of intestinal bacterial flora by using fecal specimens was conducted by TechnoSuruga Laboratory (Shizuoka, Japan) with the terminal restriction fragment length polymorphism method [16]. In brief, frozen fecal specimens were suspended, and DNA was extracted.

PCR was performed using total fecal DNA and primers of *Escherichia coli* positions 516 to 532 and positions 1510 to 1492. The resulting 16S rDNA amplicons were treated with *Bsl* l, and digested products were fractionated using an automated sequence analyzer in the DNA analysis software Gene Mapper. Because the apparent size of identical terminal restriction fragments can vary over a range of 1–3 bp, major fragments similar in size to 1–3 bp were summarized to operational taxonomic units.

### Statistical analysis

Results are expressed as means ± standard deviation for parametric data and medians for non-parametric data. All parametric data were compared using the Tukey method, and non-parametric data were compared by the Steel-Dwass method. Data were considered to be statistically significant at P<0.05.

## Results

### Body weight and biochemical analysis

**Figure 1:**
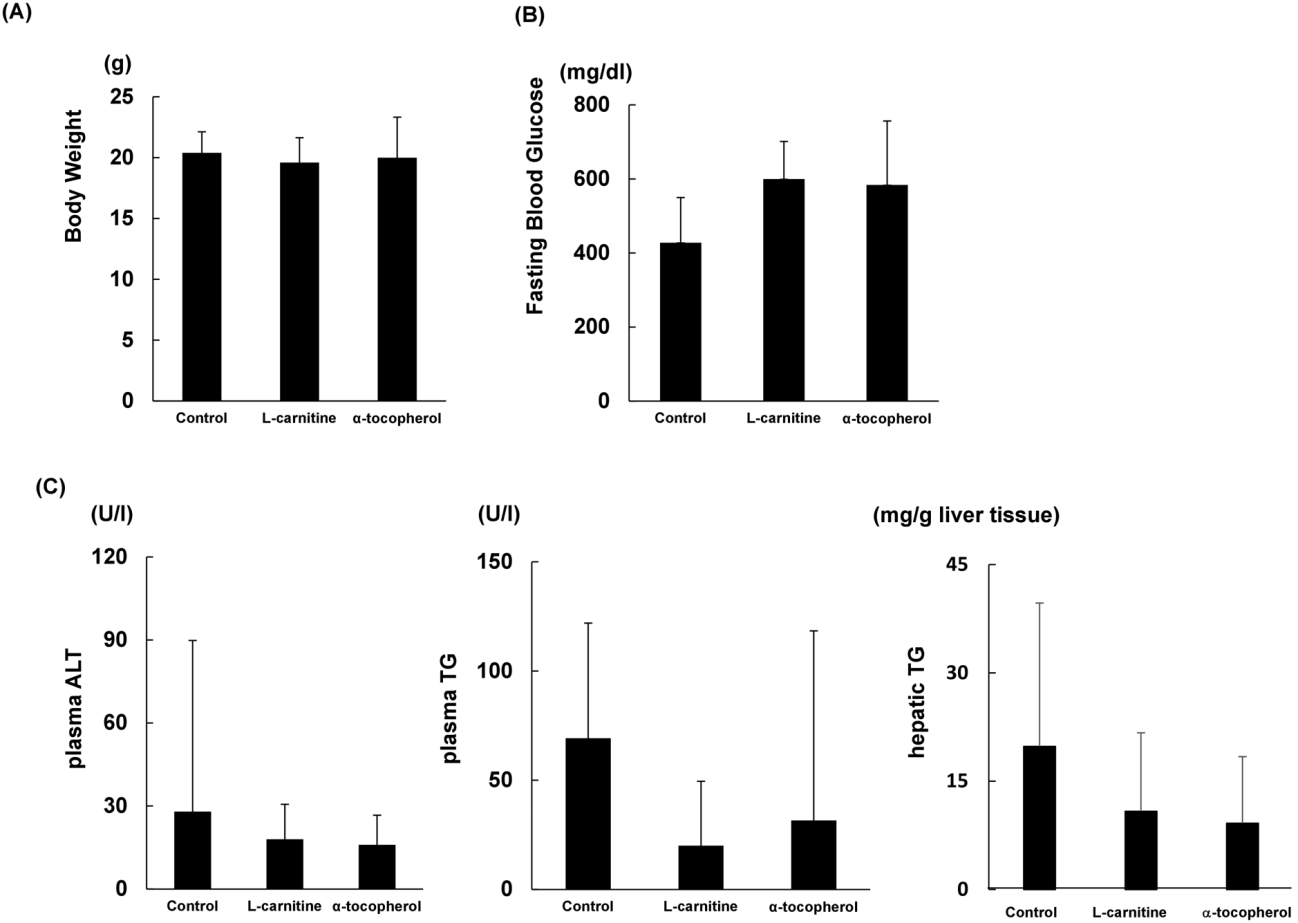
Body weight, plasma and hepatic biochemical levels. (A) Body weight of the three experimental groups. Eight-week-old male STAM mice were divided into three experimental groups and fed for 4 weeks as follows: 1) high-fat diet (HFD) (control group); 2) HFD mixed with 0.28% L-carnitine (L-carnitine group); and 3) HFD mixed with 0.01% α-tocopherol (α-tocopherol group). After 4 weeks, mice were weighed. (B) Fasting blood glucose levels of the three experimental groups. (C) Plasma biochemical findings of experimental groups. (D) Hepatic triglyceride levels of experimental groups. Data are expressed as mean ± standard deviation (SD).

There were no significant differences in body weight among the three groups (Fig. 1A). Fasting blood glucose levels were very high because pancreatic beta cells were destroyed by the streptozotocin treatment in this model (Fig. 1B). Serum alanine aminotransferase levels were slightly lower in the L-carnitine and α-tocopherol groups, serum and hepatic triglyceride levels were slightly lower in the treatment groups, although neither of these differences was statistically significant (Fig. 1C).

### L-carnitine and α-tocopherol resulted in improved NASH histology

**Figure 2:**
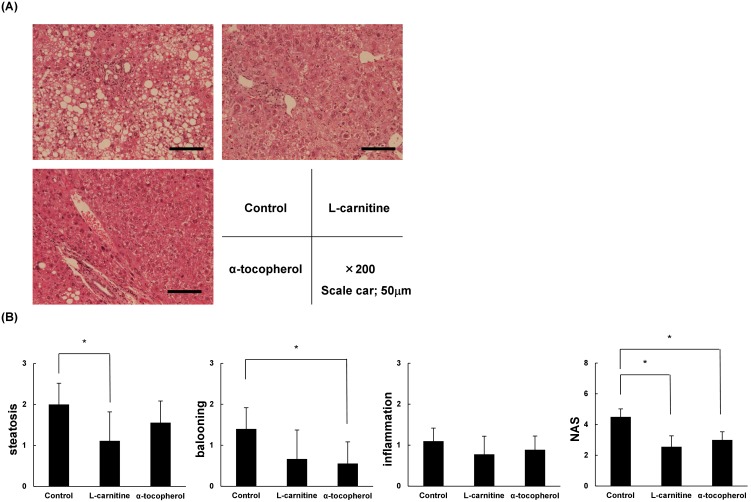
Liver histological findings. (A) Representative H&E-stained liver sections are shown. (B) Non-alcoholic fatty liver disease activity scores (NAS) for mouse liver specimens of the three experimental groups. Steatosis, inflammation, and hepatocyte ballooning were categorized, and the sum of these scores was designated as NAS. Data are expressed as means ± SD. *P<0.05.

As shown in Figure 2A, the control group developed hepatocyte steatosis, ballooning, and scattered inflammatory cell infiltration at 12 weeks. Hepatocyte steatosis was clearly reduced in the L-carnitine group at 12 weeks, and hepatocyte ballooning was clearly reduced in the α-tocopherol group at 12 weeks. NAFLD activity score was significantly lower in the L-carnitine and α-tocopherol groups (Fig. 2B).

### L-carnitine Induced strong reduction of oxidative stress in liver

**Figure 3:**
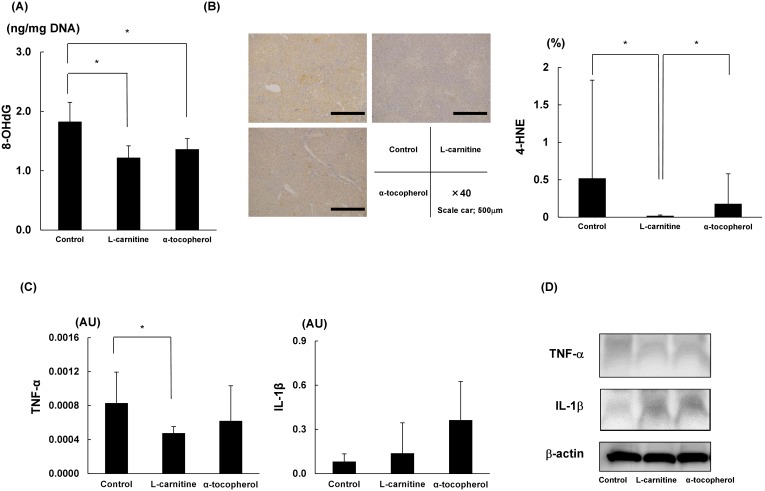
Assessment of oxidative stress and inflammation in the liver. (A) Concentrations of 8-OHdG in the liver. (B) Representative immunohistochemical staining for 4-HNE in STAM mouse liver tissue. The intensity of 4-HNE was calculated by computerized image analysis with Olympus cellSens imaging software. (C) Results of quantitative real-time PCR assay to detect TNF-α and IL-1β mRNA levels are shown. (D) Western blotting analysis of the hepatic extracts was performed with each antibodies. Data are expressed as means ± SD. *P<0.05. 8-OHdG, 8-hydroxy-deoxyguanosine; 4-HNE, 4-hydroxynonenal; TNF-α, tumor necrosis factor alpha; IL-1β, interleukin-1β.

The concentration of 8-OHdG in the liver was significantly reduced in the L-carnitine and α-tocopherol groups (Fig. 3A). The immunohistochemical staining intensity of 4-HNE was significantly decreased in the L-carnitine group (Fig. 3B), and mRNA levels of TNF-α, but not IL-1β, were significantly down-regulated in the L-carnitine group (Fig. 3C). The protein expression levels examined with Western blot analysis indicated the same pattern (Fig. 3D).

### L-carnitine and α-tocopherol upregulated mitochondrial transport-related and antioxidant defensive system-related gene expression in liver

**Figure 4:**
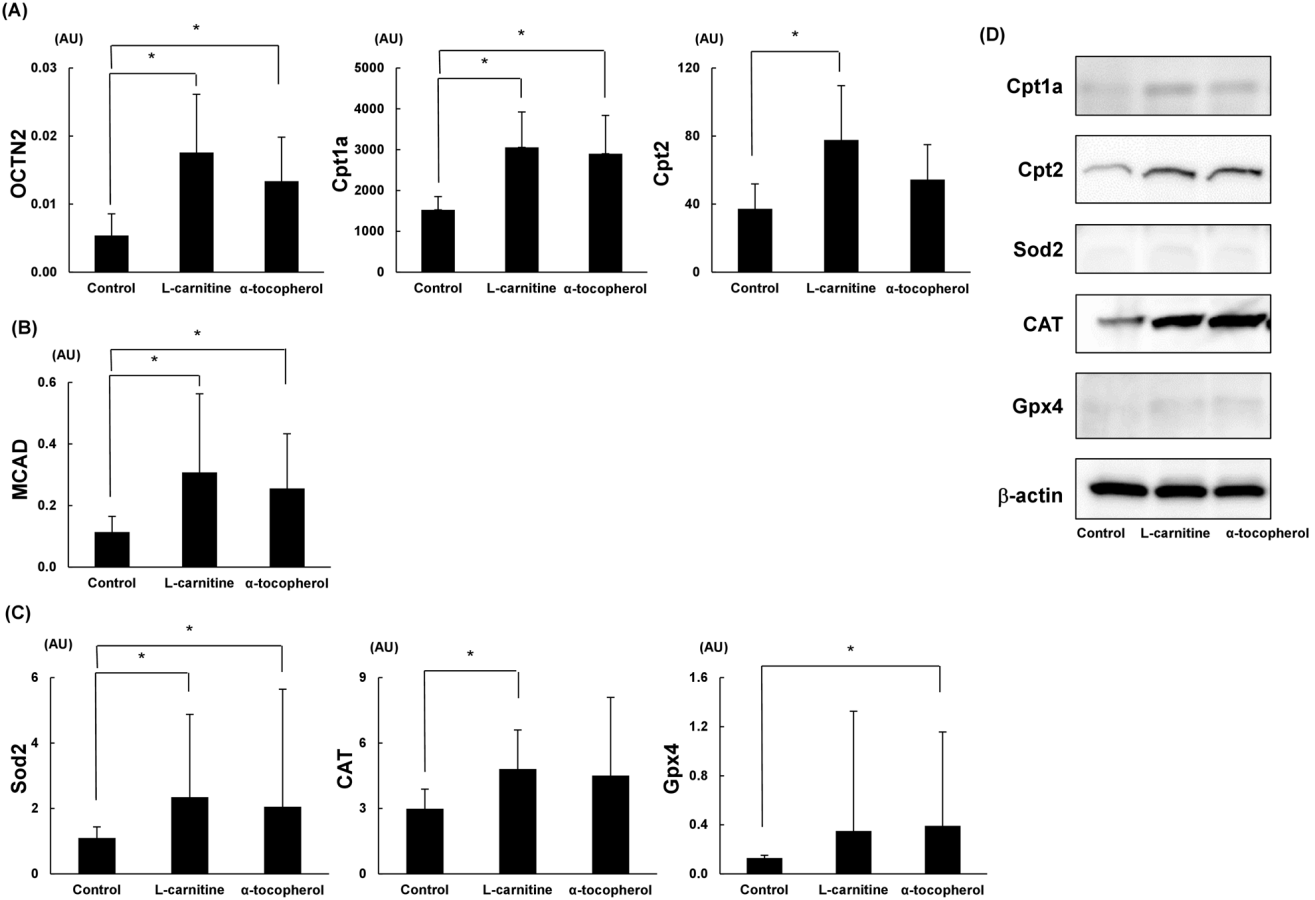
Quantitative real-time PCR results and Western blotting results for expression of hepatic mitochondrial pathway-related genes. (A) mRNA levels of L-carnitine transport-related gene OCTN-2 and long chain fatty acid transport-related genes Cpt1a and Cpt2 were analyzed. (B) mRNA levels of mitochondrial β-oxidation-related gene MCAD were measured. (C) mRNA levels of antioxidant system-related genes Sod2, CAT, and Gpx1 were analyzed. Data are expressed as means ± SD. (D) Western blotting was performed with the following antibodies directed to Cpt1a, Cpt2, Sod2, CAT and Gpx4. β-actin was used as a loading control. *P<0.05. OCTN, organic cation/carnitine transporter; Cpt, carnitine palmitoyltransferase; MCAD, medium chain acyl CoA dehydrogenase; Sod, superoxide dismutase; CAT, catalase; Gpx, glutathione peroxidase.

mRNA levels of the L-carnitine transport-related gene OCTN2 were significantly up-regulated in the L-carnitine and α-tocopherol groups. mRNA levels of LCFAs transport-related gene Cpt1a were significantly up-regulated in the L-carnitine and α-tocopherol groups, and Cpt2 was significantly up-regulated in the L-carnitine group (Fig. 4A). mRNA levels of mitochondrial β-oxidation-related gene MCAD were significantly up-regulated in the L-carnitine and α-tocopherol groups (Fig. 4B).

mRNA levels of Sod2 were significantly up-regulated in the L-carnitine and α-tocopherol groups. mRNA levels of CAT were significantly up-regulated in the L-carnitine group, and mRNA levels of Gpx4 were significantly up-regulated in the α-tocopherol group (Fig. 4C). The protein expression levels examined with Western blot analysis indicated the same pattern (Fig. 4D).

### α-tocopherol resulted in iron transport-related DMT-1 gene expression in liver

**Figure 5:**
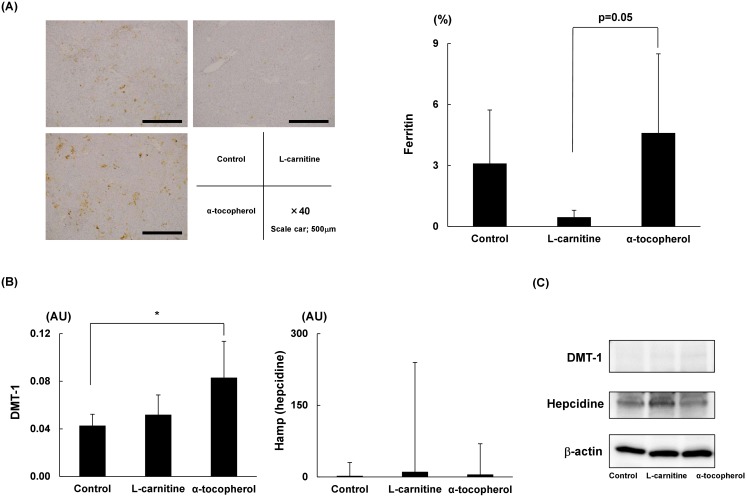
Iron metabolism related pathway analysis. (A) Representative immunohistochemical staining for ferritin of STAM mouse liver tissue. Intensity of ferritin was calculated by computerized image analysis using Olympus cellSens imaging software. (B) mRNA levels of iron uptake-related hepcidin coding gene Hamp and iron transport-related gene DMT-1 were analyzed. Data are expressed as means ± SD. (C) Liver extracted proteins were analyzed with Western blotting with antibodies directed to DMT-1 and Hamp (hepcidine). *P<0.05. DMT, divalent metal transporter; Hamp, hepcidin coding gene.

The immunohistochemical staining intensity of ferritin was significantly increased in the α-tocopherol group (Fig. 5A). mRNA levels of DMT-1 were significantly up-regulated in the α-tocopherol group. mRNA levels of Hamp (hepcidin) were not significantly different among the three groups (Fig. 5B). We next examined the protein expression levels of them using Western blot analysis indicating the same pattern.

### L-carnitine resulted in increased mitochondrial function-related metabolites in NASH

When compared with the BL6-control group, levels of 38 metabolites were significantly higher, and levels of 18 metabolites were significantly decreased in the control group (Table 1). When compared with the control group, levels of carnitine were significantly higher in the L-carnitine group. Levels of several metabolites related to the tricarboxylic acid cycle, urea cycle, and antioxidant pathway were increased in the L-carnitine group when compared with the control group. In the α-tocopherol group, several antioxidant- and urea cycle-related metabolites were also significantly higher, although to a lesser extent than in the L-carnitine group (Table 2).

**Table 1:** Metabolomic analysis (Control/BL6-Control).

Pathway	Metabolite	Control vs. BL6 control		
		Ratio¶	*p*-value||	
β-Alanine metabolism	3-Ureidopropionic acid	4.4	0.003	**
ATP-binding cassette (ABC) transporter	Putrescine	4.6	0.011	*
Folate biosynthesis	5,6,7,8-Tetrahydrobiopterin	3.9	0.004	**
Citrate cycle (TCA cycle)	Malic acid	2.8	0.018	*
Inositol phosphate metabolism	myo-Inositol 1-phosphate	2.2	0.004	**
Inositol phosphate metabolism	myo-Inositol 3-phosphate	2.2	0.004	**
Nicotinate and nicotinamide metabolism	1-Methylnicotinamide	2.2	0.028	*
β-Alanine metabolism	β-Alanyl-L-lysine(β-Ala-Lys)	2.1	0.018	*
Butanoate metabolism	2-Hydroxyglutaric acid	1.8	0.043	*
Ascorbate and aldarate metabolism	Ascorbic acid	1.9	0.028	*
ATP-binding cassette (ABC) transporter	Glutamate	1.8	0.006	**
ATP-binding cassette (ABC) transporter	Glutathione (GSH)	1.5	0.020	*
Glutathione metabolism	γ-glutamylcysteine (γ-Glu-Cys)	1.5	0.047	*
Ascorbic acid and aldaric acid metabolism	Threonic acid	1.4	0.016	*
Glycerophospholipid metabolism	Ethanolamine phosphate	1.4	0.025	*
ATP-binding cassette (ABC) transporter	Aspartic acid	1.4	0.029	*
Synthesis and degradation of ketone bodies	3-Hydroxybutyric acid	1.4	0.040	*
Citrate cycle (TCA cycle)	Succinic acid	1.3	0.015	*
ATP-binding cassette (ABC) transporter	Lysine	0.7	0.001	***
Ascorbic acid and aldaric acid metabolism	Ribulose 5-phosphate	0.7	0.002	**
Nicotinate and nicotinamide metabolism	Nicotinamide	0.7	0.001	***
ATP-binding cassette (ABC) transporter	Leucine	0.7	0.007	**
Urea cycle	Creatinine	0.7	0.007	**
Glycine, serine and threonine metabolism	Homoserine	0.7	0.014	*
ATP-binding cassette (ABC) transporter	Valine	0.7	0.032	*
Zeatin biosynthesis	5′-Deoxy-5′-methylthioadenosine	0.7	0.044	*
Amino sugar and nucleotide sugar metabolism	Glucose 6-phosphate	0.7	0.047	*
ATP-binding cassette (ABC) transporter	Isoleucine	0.6	0.000	***
Purine metabolism	Inosine	0.6	0.005	**
Urea cycle	Ornithine	0.6	0.005	**
Glycine, serine and threonine metabolism	N,N-Dimethylglycine	0.6	0.009	**
Cysteine metabolism	Glutathione (GSSG)_divalent	0.6	0.011	*
Glutathione metabolism	NADP+	0.6	0.011	*
ATP-binding cassette (ABC) transporter	Glycerol 3-phosphate	0.6	0.011	*
ATP-binding cassette (ABC) transporter	Threonine	0.6	0.013	*
Pyrimidine metabolism	Thymidine	0.6	0.016	*
Pyrimidine metabolism	Cytidine monophosphate (CMP)	0.6	0.042	*
ATP-binding cassette (ABC) transporter	Thiamine	0.6	0.043	*
ATP-binding cassette (ABC) transporter	Glycine	0.5	0.000	***
Thiamine metabolism	Thiamine phosphate	0.5	0.000	***
Purine metabolism	Guanosine	0.5	0.009	**
β-Alanine metabolism	β-Alanine	0.5	0.010	*
β-Alanine metabolism	Carnosine	0.5	0.014	*
Histidine metabolism	3-Methylhistidine	0.5	0.027	*
Purine metabolism	ADP-ribose	0.5	0.034	*
Lysine degradation	γ-Butyrobetaine	0.4	0.000	***
Pyruvate metabolism	Phosphoenolpyruvic acid	0.4	0.000	***
Glycine, serine and threonine metabolism	Betaine aldehyde_+H2O	0.4	0.001	**
β-Alanine metabolism	Gamma-aminobutyric acid (GABA)	0.4	0.003	**
Purine metabolism	Guanine	0.4	0.004	**
Pyrimidine metabolism	Cytidine	0.4	0.006	**
ATP-binding cassette (ABC) transporter	Serine	0.4	0.007	**
Glyoxylate and dicarboxylate metabolism	3-Phosphoglyceric acid	0.4	0.008	**
Glycerophospholipid metabolism	Phosphorylcholine	0.3	0.015	*
Arginine and proline metabolism	5-Aminovaleric acid	0.2	0.003	**
Histidine metabolism	Ergothioneine	0.0	0.024	*

¶ ; ratio of left divided by right.

|| ; p-value: ***p<0.001, **p<0.01, *p<0.05.

**Table 2:** Metabolomic analysis (L-carnitine/Control, α-tocopherol/Control, L-carnitine/α-tocopherol).

Pathway	Metabolite	L-carnitine / Control			α-tocopherol / Control			L-carnitine / α-tocopherol		
		Ratio ¶	*p*-value ||		Ratio ¶	*p*-value ||		Ratio ¶	*p*-value ||	
Alanine and aspartate metabolism	Argininosuccinic acid	1.7	0.002	**	1.3	0.040	*	1.3	0.006	**
ATP-binding cassette (ABC) transporter	Carnitine	1.7	0.005	**	1.2	0.152		1.4	0.007	**
Cysteine metabolism	Glutathione (GSSG)_divalent	1.6	0.013	*	1.6	0.019	*	1.0	0.787	
Vitamin B6 metabolism	Pyridoxal	1.5	0.002	**	1.3	0.101		1.2	0.121	
Glyoxylate and dicarboxylate metabolism	Tartaric acid	1.5	0.042	*	1.6	0.488		0.9	0.895	
Thiamine metabolism	Thiamine phosphate	1.4	0.004	**	1.2	0.028	*	1.2	0.022	*
Citrate cycle (TCA cycle)	Malic acid	1.4	0.004	**	1.2	0.628		1.2	0.473	
Fatty acid biosynthesis	Lauric acid	1.4	0.005	**	1.1	0.682		1.3	0.170	
Fatty acid biosynthesis	Decanoic acid	1.4	0.011	*	1.0	0.778		1.4	0.040	*
Propanoate metabolism	2-Hydroxybutyric acid	1.4	0.033	*	1.3	0.296		1.1	0.806	
Glycerophospholipid metabolism	Glycerol 3-phosphate	1.4	0.039	*	1.2	0.355		1.1	0.497	
Methane metabolism	5-Methyltetrahydrofolic acid	1.3	0.009	**	1.0	0.786		1.4	0.038	*
Citrate cycle (TCA cycle)	Fumaric acid	1.3	0.043	*	1.2	0.620		1.1	0.593	
Valine, leucine and isoleucine degradation	Valine	1.6	0.167		1.5	0.021	*	1.0	0.843	
Taurine and hypotaurine metabolism	Taurocyamine	1.2	0.317		1.3	0.017	*	0.9	0.486	
Urea cycle	Urea	1.5	0.056		1.2	0.041	*	1.2	0.142	
Urea cycle	Citrulline	1.4	0.132		0.9	0.563		1.6	0.042	*
Purine metabolism	ADP-ribose	0.5	0.023	*	1.0	0.870		0.5	0.125	
Glycolysis / Gluconeogenesis	Glucose 1-phosphate	0.7	0.015	*	0.8	0.111		0.9	0.231	
Folate biosynthesis	5,6,7,8-Tetrahydrobiopterin	0.7	0.027	*	0.8	0.079		0.9	0.264	
Alanine and aspartate metabolism	Alanine	0.7	0.030	*	0.9	0.327		0.8	0.262	

¶ ; ratio of left divided by right.

|| ; p-value: ***p<0.001, **p<0.01, *p<0.05.

### α-tocopherol induced intestinal microbiome changes

**Figure 6:**
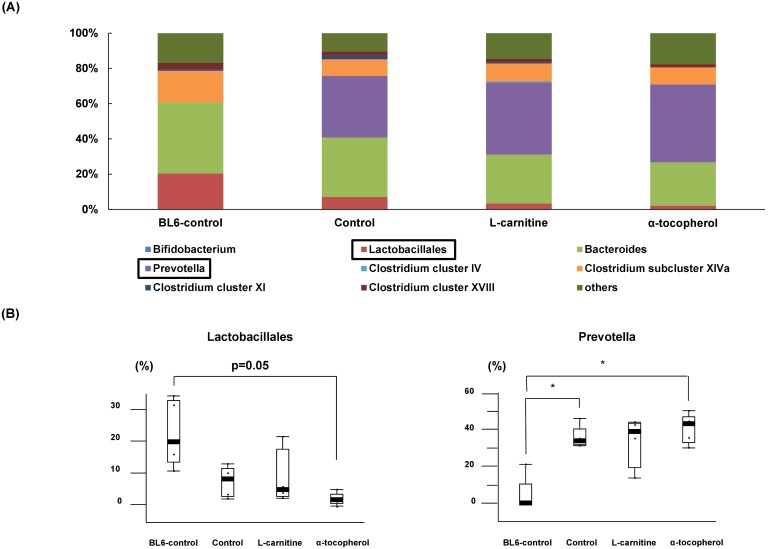
Intestinal microbiome analysis. (A) The 100% stacked column chart of microbiome from intestinal feces. (B) Representative percentages of bacterial species in experimental groups. *P<0.05.

The intestinal bacterial flora in the NASH model mice was characterized by less *Lactobacillales* and more *Prevotella* than in healthy controls (Fig. 6A). These differences were more evident in the α-tocopherol group but less evident in the L-carnitine group.

### L-carnitine reduced hepatic tumorigenesis

**Figure 7:**
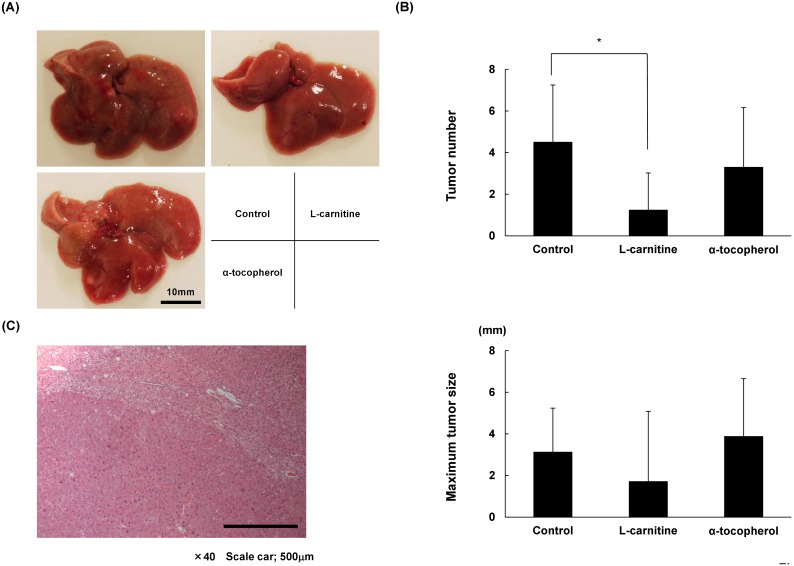
Effects of drugs on preventing hepatocarcinogenesis. (A) Control group developed numerous tumors on the liver surface, while groups receiving drug treatments developed fewer tumors. (B) Histological findings showed that the tumors are hepatocellular carcinoma. (C) Number of tumors was significantly lower in the L-carnitine group. In the α-tocopherol group, the average tumor number and size were not reduced. Data are expressed as means ± SD. *P<0.05.

Liver tumors were found in all mice in each STAM group at 16 weeks (Fig. 7A). Histological findings showed that the tumors were HCC (Fig. 7B). The L-carnitine group developed fewer tumors. There were no significant differences in tumor size among the three groups (Fig. 7C).

## Discussion

The present results confirmed that L-carnitine increased hepatic expression of genes related to LCFAs transport, mitochondrial β-oxidation, and antioxidant enzymes following suppression of hepatic oxidative stress markers and inflammatory cytokines in NASH. Furthermore, L-carnitine reduced NASH-related hepatic tumorigenesis in mouse models. α-tocopherol resulted in NASH improvement in the same manner. However, it increased periodontitis-related microbiotic change and hepatic iron transport-related gene expression, and ultimately led to more severe hepatocarcinogenesis. These results indicate that L-carnitine represents a simple and novel therapeutic strategy for NASH.

Numerous drugs have been tested for the potential to alleviate fatty liver and NASH. These treatments have diverse pharmacological activities such as improvement of insulin sensitivity, stimulation of lipid oxidation, as well as reduction of *de novo* lipogenesis, oxidative stress, and inflammation that are characteristics of NASH [17]. Among these drugs, the antioxidant drug vitamin E is the first-line treatment recommendation for NASH.

ROS are widely accepted as a source of oxidative stress that appears to be responsible for the initiation of necroinflammation. ROS are generated during the metabolism of free fatty acids in microsomes, peroxisomes, and particularly in mitochondria. Most of the electrons provided to the mitochondrial respiratory chain migrate along this chain to finally reach cytochrome c oxidase, where they safely combine with oxygen and protons to form water [18]. However, some of these electrons leak to form the superoxide anion radical. This radical can then be dismutated by Sod2 into hydrogen peroxide, which is normally detoxified into water by Gpxs and CAT [19]. Thus, most mitochondrial ROS are usually detoxified, and residual ROS serve as signaling molecules. Physiologically low levels of ROS are involved in necessary vital cellular processes, indicating that an adequate control of oxidative stress and balance of oxidative and anti-oxidative responses is important [20]. Excess superoxide could be generated within injured mitochondria through electron leakage, and the resulting excess of superoxide would be converted to hydrogen peroxide by Sod2. Gpxs or CAT can metabolize hydrogen peroxide to non-toxic H_2_O, but the Fenton and/or Haber-Weiss reactions mediated by iron generate highly reactive toxic ROS, hydroxyl radicals. Levels of iron are elevated in NASH, which is an inducer of oxidative stress, and reduced iron levels result in fair outcomes for patients with chronic liver diseases [21].

L-carnitine supplementation in NASH patients greatly improved glucose plasma levels, lipid profiles, and histological manifestations [22]. Furthermore, L-carnitine ameliorated fatty liver in high-calorie diet/streptozotocin-induced type 2 diabetic mice by improving mitochondrial function [23]. We assumed that L-carnitine may alter not only the LCFA uptake into mitochondria, but also the activity of the ROS-scavenging antioxidant enzymes in NASH model mice. The present data showed that L-carnitine reinforced the mitochondrial β-oxidation and the activity of the key ROS-scavenging antioxidant enzymes such as Sod2 and CAT without an increase in oxidative stress. In addition, L-carnitine has recently been shown to exhibit ammonia reduction in hepatic encephalopathy patients and improvement of fatigue, which reflects the wide pharmacological effects of L-carnitine on hepatic and muscular mitochondrial function recovery [24]. We were not able to demonstrate such effects of L-carnitine in our model, as we did not examine plasma ammonia levels or physical scores that reflect hepatic encephalopathy and related effects.

The role of α-tocopherol in the treatment of NASH is based on its activity as a free-radical scavenger. α-tocopherol is a chain-breaking antioxidant in free-radical reactions, which is an important step in lipid peroxidation and membrane stabilization [25]. Animal studies have shown that α-tocopherol improves fibrosis, reduces mitochondrial lipid peroxidation, and corrects oxidative stress in animal models of liver disease associated with oxidative injury [26]. However, the life-long administration of α-tocopherol to animals exposed to cold or warm stress resulted in a significantly shortened life span [27]. In humans, several randomized controlled trials have indicated a potential role for vitamin E supplementation in NAFLD [28], [29]. These studies included biochemical data and liver histological assessment, but they lasted for only several years. Many cerebrovascular disease studies have investigated the effects of vitamin E. A meta-analysis of the effect of vitamin E on stroke revealed a 10% reduction in ischemic stroke accompanied by a 22% increase in hemorrhagic stroke [30]. Furthermore, meta-analysis revealed that all-cause mortality with vitamin E and vitamin A supplementation was worse than for controls [31]. Our data showed that α-tocopherol increased mitochondrial β-oxidation-related enzyme gene expression without increased oxidative stress or altered activity of key ROS-scavenging antioxidant enzymes such as Sod2 and Gpx4. Overall, the trend was stronger in the L-carnitine group, with the exception that the expression of the iron transport-related gene DMT-1 was higher in the α-tocopherol group than in the L-carnitine group.

Iron is an essential metal for all organisms and plays a crucial role in a variety of cellular functions in metabolism, growth, and differentiation, such as oxygen transport and storage, energy production, cell cycle, and DNA synthesis [32]. However, excess cellular iron is toxic, resulting in hydroxyl radical production via the Fenton reaction. Ferritin is an important hub for iron metabolism. Ferritin sequesters iron during conditions of iron excess and releases iron during conditions of iron scarcity [33]. In the present study, intrahepatic ferritin was expressed at higher levels in the α-tocopherol group than in the L-carnitine group, while the expression of important iron metabolism-related genes such as hepcidin showed no difference. These results suggest that the higher expression of ferritin in the α-tocopherol group was not due to simple iron load but that oxidative stress or inflammatory cytokines induced ferritin expression. In human cirrhotic livers, DMT-1 and ferritin mRNA are more highly expressed than in control donor livers, possibly due to an inflammatory or stress-responsive pathway, while hepcidin expression showed no difference [34]. While the exact mechanism of why such differences are seen in human cirrhosis is unknown, cirrhotic conditions in the liver might induce changes in iron metabolism. As inflammatory cytokines such as interleukin-1β showed no difference between the two drug groups in our model, an aberrant oxidative stress condition between these two drugs might be an important issue.

NASH is an accepted risk factor for hepatic carcinogenesis, but the precise mechanism contributing to hepatocarcinogenesis remains unclear. Gut microbiota changes are involved in NASH progression [35]. Recently, obesity-induced gut microbiota changes such as *Bacteroides* reduction have been shown to increase the levels of deoxycholic acid, a gut bacterial metabolite that causes DNA damage resulting in increased incidence of hepatocellular carcinoma [36]. In our non-obese NASH model, *Prevotella* (*Bacteroides*) proliferated more than normal controls, while it has been accepted to be less common in obesity patients. *Prevotella* is correlated with periodontal diseases or upper respiratory tract infection. In our present results, α-tocopherol showed the strongest effect on benign *Lactobacillus* reduction and *Prevotella* (*Bacteroides*) proliferation, which might be one reason for the unfavorable outcome.

Oxidative stress has been shown to be involved in hepatocarcinogenesis with telomerase activation or increased neovascularization [37], [38]. One oxidative stress marker, 8-OHdG, is increased in NASH-related hepatocellular carcinoma patients [39]. Current human therapeutic drug trials on NASH revealed only short-term improvement in laboratory data and NASH histology but no data in the long-term outcome of hepatocarcinogenesis or mortality. NASH mouse models developing HCC must be a good marker to reveal the long-term effect of treatments. The STAM NASH model is a promising model to show NASH-related hepatocarcinogenesis, but lean body with low adipose fat and low insulin levels exhibits a different course than human NASH hepatocarcinogenesis. There are several diabetic or obesity models that exhibit NAFL or NASH, but typically show a low incidence of HCC. We believe that such models reflect only NAFL or non-advancing NASH, which are not treatment targets; thus we selected this model in our experiments. The hepatic tumor number was reduced in the L-carnitine group. Differences between the L-carnitine and α-tocopherol groups, such as 4-HNE expression, iron transport-related DMT-1 gene expression, mitochondrial pathway-related metabolomics profiles, and intestinal microbiome patterns might be involved in anti-hepatocarcinogenesis mechanisms. Oxidative stress is accepted as carcinogenic, but antioxidant treatment has been shown to exacerbate cancers. Antioxidants might permit cancer cells to transform into the stem cell-like cancer cells that have powerful antioxidative properties and are thus anti-apoptotic [40]. L-carnitine has histone deacetylase-inhibiting potential and thus shows anti-tumor activity against HCC cell lines and mouse models, which might be one reason for the better outcome of the L-carnitine group [41].

In conclusion, we confirmed that L-carnitine improves the hepatic expression of genes related to LCFAs transport, mitochondrial β-oxidation, and metabolites following decreased oxidative stress. Furthermore, L-carnitine improves NASH-related hepatocarcinogenesis in a mouse model. These results indicate that the mitochondrial function modifier L-carnitine provides a simple and novel therapeutic strategy for NASH.
